# Description of a New Species of Mountain Midges (Diptera, Deuterophlebiidae) from Xinjiang, China ^†^

**DOI:** 10.3390/insects16090965

**Published:** 2025-09-15

**Authors:** Xin Wang, Minghui Gao, Xinyang Li, Rui Han, Jiayang Feng, Wei Guo

**Affiliations:** Xinjiang Key Laboratory for Ecological Adaptation and Evolution of Extreme Environment Organisms, College of Life Sciences, Xinjiang Agricultural University, Urumqi 830000, China; 18146913651@163.com (X.W.); gmhxxzj@xjau.edu.cn (M.G.); 15599773075@163.com (X.L.); lip546686@163.com (R.H.); 18030472729@163.com (J.F.)

**Keywords:** Deuterophlebiidae, pupae, COI, description, *Deuterophlebia shawanensis* sp. nov.

## Abstract

The family Deuterophlebiidae is considered to be the most plesiomorphic Diptera family, with a striking morphology and life history. In this study, we provide detailed descriptions and figures of a new species from Northwestern China: *Deuterophlebia shawanensis* sp. nov., along with genetic distances of COI sequences among the eight known Chinese species of *Deuterophlebia* Edwards, 1922. The genetic distance from others is greater than 0.1. In terms of morphology, the new species could also be distinguished from others by the absence of spines in the mesothorax at the pupal stage and the outward protrusion of the second dorsal view of the mesothorax. As a result, China is currently the country with the highest *Deuterophlebia* species diversity.

## Simple Summary

Deuterophlebiidae, one of the most peculiar insects of the order Diptera, has interesting morphological and biological characteristics; its larvae and pupae prefer to inhabit smooth stones in fast-flowing streams and adults have extremely long antennae and a lifespan of only a few hours. Research on it began a century ago with 21 species being reported so far; in recent years, species have been reported in China, although they have only been found in Southern China. We carefully classified an unknown species collected in Shawan, Xinjiang, China, and optimized data on diversity and genetic distance between other species in China.

## 1. Introduction

The species of Deuterophlebiidae, also named the mountain midge, is a monotypic family in the order Diptera, which has a peculiar appearance and unique living habits. The first-ever fossil of mountain midges (Diptera: Deuterophlebiidae) are described from Mid-Cretaceous Burmese amber [1]. They are firmly nested within Psychodomorpha as a sister to Hennigmatidae, resolving their contested position in Diptera evolution [1]. The larvae of Deuterophlebiidae are peculiarly shaped, divided into eight segments, with distinctly reversible crocheted pseudopods perched on smooth stones; the pupae are distinctly streamlined, and the adult worms usually degenerate their mouthparts and mate at sunrise, with the male being dead immediately after that, while the female sheds her wings and returns to the waterbody to lay eggs [2,3,4].

Their immature stage is highly specialized and they usually inhabit cold, fast-flowing rapids [5]. The first species was found in the mountains of Kashmir, India [6]. By 2022, there were 14 named species in this family around the world, with 8 of them being from the Palearctic region [5,7,8], and the rest being located in the New Arctic Domain [2,9,10,11,12]. Among them, *Deuterophlebia nipponica* Kitakami, first collected by Kitakami in a fast-flowing stream near Kyoto, Japan in 1926 [5,13], has not been rediscovered ever since, and has since been listed as an endangered species in Japan. In 2022, five new species were reported in Southwestern and Eastern China, enriching the diversity and geographic distribution of domestic and global species in the family; these species are *D. sinensis*, *D. wuyiensis*, *D. yunnanensis*, *D. alata*, and *D. acutirhina* [4]. Because they were collected at the same place and time, *D. sinensis* and *D. wuyiensis* were also considered to be polymorphic or highly alienated populations [4]. In 2023, two new species (*D. pseudopoda*, *D. pachychaeta*) were reported in Yunnan Province, Southwestern China, giving the country the greatest diversity of Deuterophlebiidae (*D. mirabilis, D. sinensis*, *D. wuyiensis*, *D. yunnanensis*, *D. alata*, *D. acutirhina, D. pseudopoda*, and *D. pachychaeta*) [13]. The biogeographic distribution and phylogenetic status of the family remains a mystery [4,5], which probably means that there are more new species in this family waiting to be found worldwide.

In the last year, we collected over one hundred larvae and pupae of *Deuterophlebia* from Xinjiang province, China. Morphological examination revealed that it differed from all previously reported species in China. We thus identified it as a new species, sequenced its COI genes, calculated the genetic distances to the known Chinese species at the molecular level, and integrated both morphological and molecular evidence to confirm its status as a new species. The results of the study indicate that there are more habitats of this species in China, and their distribution maps have been updated.

## 2. Materials and Methods

During an aquatic survey, we accidentally collected some samples (larvae and pupae) belonging to *Deuterophlebia*, which were sent for further study. Samples are collected mainly through the following methods: larval samples are collected from the surface of stones, directly, or with benthic nets by filtrating the water; samples of pupae are picked from the smooth surface of the stones in the current with tweezers [4]. The specimens we retrieved were sent back to the laboratory and examined under a stereomicroscope (NIKON SMZ25, Nikon, Tokyo, Japan) [13], observed, and photographed. The terminology mainly follows that of Courtney (1994) [5]. All samples were stored in absolute ethanol and formaldehyde solution and stored in Xinjiang Key Laboratory for Ecological Adaptation and Evolution of Extreme Environment Organisms, College of Life Sciences, Xinjiang Agricultural University.

We conducted morphological differentiation on over one hundred collected samples and randomly selected 20 groups, each consisting of 3 samples for molecular analysis. To confirm and identify the larvae and pupae, total genomic DNA was extracted from the abdomen and head of the specimens using an Animal Genomic DNA Kit (Sangon Biotech, Shanghai, China). The extracted DNA was sent to Sangon Biotech (Shanghai, China) for further sequencing. The mitochondrial gene cytochrome c oxidase subunit I fragment was PCR-amplified using the Premix Taq (Sangon Biotech, Shanghai, China) with forward primer LCO1490 (5′-GGTCAACAAATCATAAAGATATTGG-3′) and reverse primer HCO2198 (5′-TAAACTTCAGGGTGACCAAAAAATCA-3′) [4,14]. PCR conditions included initial denaturation at 94 °C for 5 mins, 40 cycles of denaturation at 94 °C for 30 s, annealing at 50 °C for 30 s, and extension at 72 °C for 40 s, with a final extension at 72 °C for 10 mins [4]. For the sequences to be detected, the homology of the sequences was determined using NCBI and DNAMAN, and the K2P genetic distance was estimated in MEGA11 [15]. All sequences with GenBank accession number and specimen information are listed in Table 1.

## 3. Results

### 3.1. Morphological Description of the Species

*Deuterophlebia shawanensis* sp. nov. (Nomeclatural author: X.W.)

**Figure 2:**
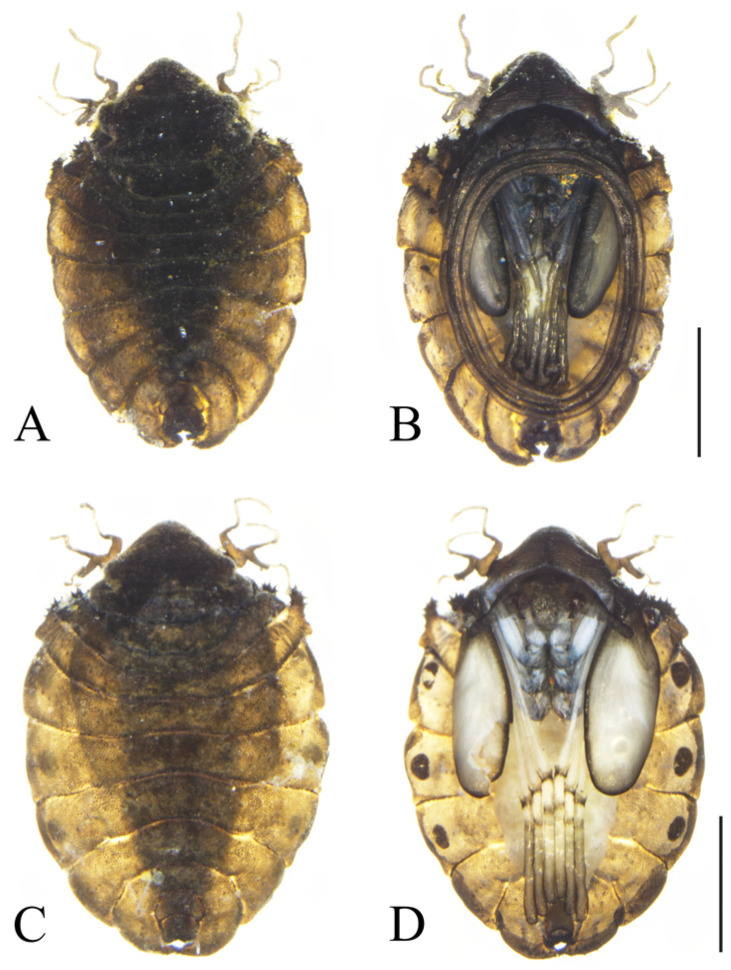
Pupae of *D. shawanensis* sp. nov.: (**A**). Male pupa (dorsal view); (**B**). Male pupa (ventral view); (**C**). Female pupa (dorsal view); (**D**). Female pupa (ventral view). Scale bars = 1.0 mm.

**Type material**: Regarding the holotype of Deuterophlebiidae *Edw.* in the first published article on the proposed family of Deuterophlebiidae [16], there are significant morphological differences compared to the species we discovered. Firstly, the male and female pupae are shorter than our specimens. Secondly, the second thoracic segment is not prominently raised and is longer than the third thoracic segment, and the gill filament length is also shorter than that of our specimens. Our specimens have no spines on the dorsal side of the head capsule, so there are clear morphological differences from the holotype (Figures 5 and 6 in Ref. [16] and Figure 2C,D). Additionally, morphological comparisons with other species are also discussed in the article. Our type materials are preserved at Xinjiang Key Laboratory for Ecological Adaptation and Evolution of Extreme Environment Organisms, College of Life Sciences, Xinjiang Agricultural University.

**Type locality**: Regarding the collection sites of our specimens, we already indicated the latitude, longitude, and elevation of the sampling locations in the article, as well as other hydrological data at the time of sampling. We believe we have described them very accurately. For specific location information, please refer to Tables 1 and 3 and Figure 4 in the article.

**Type material deposition place**: The sampled rivers include a variety of substrate types such as silt, coarse sand, and gravel, and the surrounding habitats consist of forests, grasslands, and shrublands.

**Figure 1:**
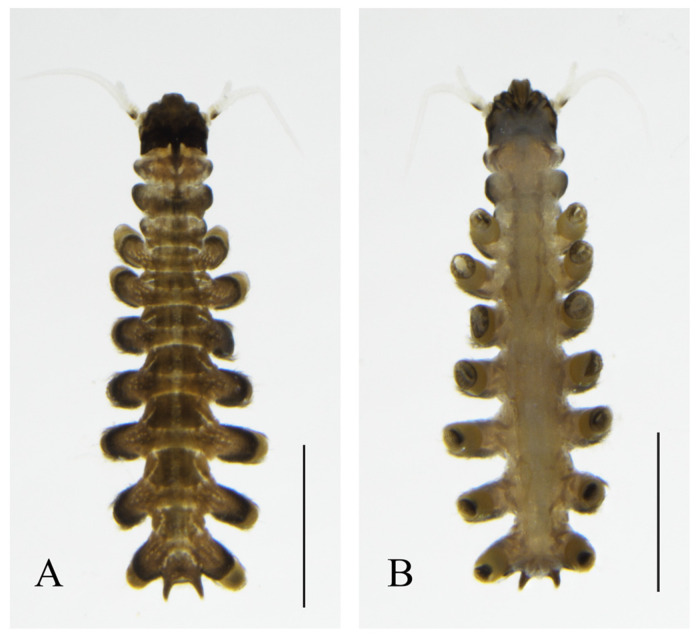
Larva of *D. shawanensis* sp. nov.: (**A**) dorsal view; (**B**) ventral view. Scale bar = 1.0 mm.

Description. **Larva.** The length of the larva is 3.07~3.10 mm (Figure 1). Refer to the information summarized by previous generations; the collected larvae are in the third age stage, and the color of the prothorax turns black. The pigment begins to precipitate and is more obvious, and the color of the mesothorax is darker than that of the prothorax. There is a dark cinnamon-colored trapezoidal pattern between each abdominal segment, and there is a white border. The base of the protrusions on both sides also becomes darker. The cephalic sac is dark cinnamon-colored, almost black at the base of the antennal axis, but relatively short.

**Figure 3:**
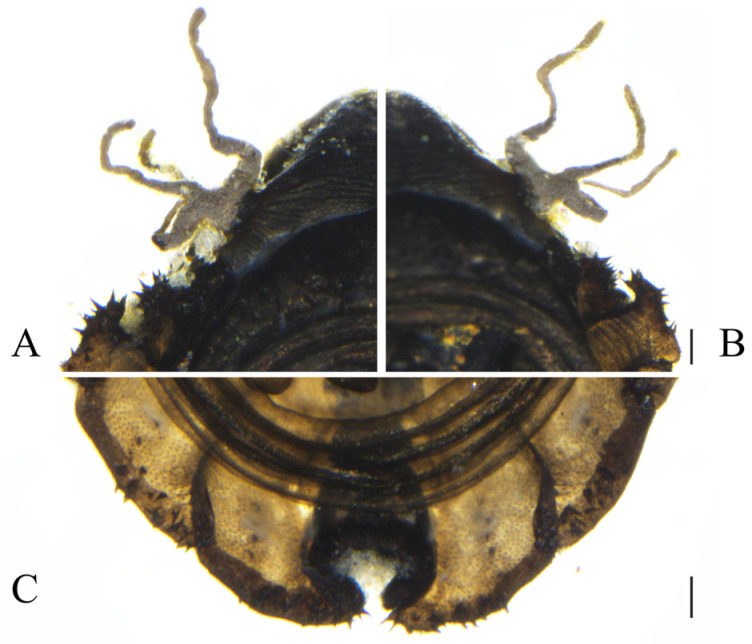
Male pupae of *D. shawanensis* sp. nov.: (**A**). The small spines (ventral view); (**B**). Gill (ventral view); (**C**). Posterior end (ventral view). Scale bars = 0.1 mm.

Description. **Male pupae.** Pupae flattened oval shaped, length 3.41~3.99 mm, width 2.14~2.56 mm (Figure 2A,B). Dorsal integument dark brown, flattened, divided into 11 segments, densely covered with brown dots. The shell is dark and dark cinnamon and the edge of the pupal is lighter, with the midline being darker. None meso- and meta-thoracic segments with paired transverse chitin bands, both sides of the mesothorax with a gill, originated from a round base. The gill has three gill filaments (different from the few four gill filaments which were reported in China), longer, and light gray in color (Figure 2A,B). These three filaments have similar shape, slender and twisted, pointing in different directions, and the gills are easy to fall. The anterior filament and second filament slightly longer than the posterior filament. The first and second abdominal segments are similar, each with a pair of spine-decorated anterolateral projections, and spines also exist on the lateral margin of segments VI and VII. However, the edge of the second abdominal segment is prominently protruding, so that the second abdominal segment is much wider than the third abdominal segment. The small spines grow on the edge of this irregularly shaped protrusion, each projection pointing forward and bearing ca. 13–20 spines, with the length of 0.03–0.07 mm, and the spines are short and slightly curved, broad at the base, and pointed at the apex. Most of the terminal spines of section VI only have one, while the body ends also have clusters of spines, with a number ranging from 2 to 9 (Figure 3C). Segment VIII shield shaped, surrounded by segments VII and IX. The antennal sheath extends from both sides and encircles the body 2.0 times, forming a large elliptical ring located on the ventral surface. Six leg pouches extend to the posterior part of the antennal ring, with a bulging top. There are three pairs of black sticky disks on the ventral margin of the third to fifth segments of the abdomen. The whole back is covered with a large number of black spots. The pupal period changes over time, and the old mature pupae are darker than the immature pupa.

Description. **Female pupae**. Pupae flattened oval shaped, length 3.23~3.83 mm, width 2.16~2.55 mm (Figure 2C,D). Female pupae are similar to males except for smaller body size, smaller mesothorax, and sexual differences on the ventral side, including thinner and shorter antennal sheaths and not expanded apex of leg sheaths. Other characteristics are similar to those of males. It is worth thinking about the sex ratio of the pupal stage, which is about 2:1 in the samples we collected. Compared with males, females are more able to see three pairs of large black spots on their abdomen, which are unique to the family Deuterophlebiidae.

### 3.2. Molecular Study

In this study, the COI gene fragment of the new species (*D. shawanensis* sp. nov.) was sequenced and analyzed alongside seven other Chinese species. We compared these in DNAMAN and estimated genetic distance in MEGA11.

The interspecific genetic distances range from 0.086 to 0.156 (Table 2). The smaller the genetic distance in the table, the more yellow the color is; at 0.086 (between *D. pachychaeta* and *D. yunnanensis*), orange is used to indicate the lowest distance. The larger the genetic distance, the greener the color is, reaching the highest at 0.156 (between *D. sinensis* and *D. acutirhina*), which is grass green. One group is under 0.10, with this difference being between *D. pachychaeta* and *D. yunnanensis*. In the table, the genetic distance between *D. shawanensis* sp. nov. and other published species in China is greater than 0.10, which may be different from other species in the phylogenetic tree.

### 3.3. Biological Notes

**Figure 4:**
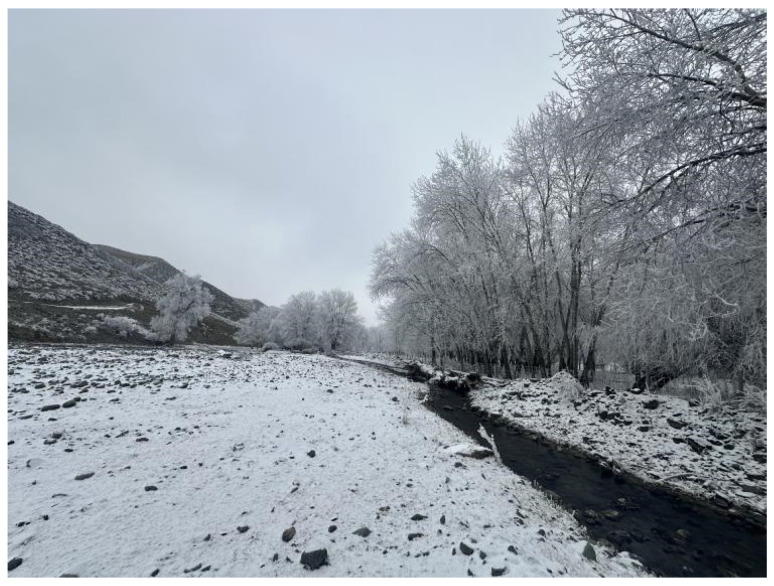
Habitat stream of *D. shawanensis* sp. nov.

The specimens were collected in Da Nangou from October to December in 2024, and the water area was about 1.5 m wide and 1.2 m deep (other hydrological data are shown in Table 3), unobstructed, with stones of various sizes in the water (Figure 4). The surrounding area is a grassland forest habitat.

Both pupae and larvae were collected at the same location. The pupae were collected in the rapids zone, and the ventral disk of the pupae was tightly attached to the surface of the stone and lived on a single stone with a large number of the Simuliidae larvae. These biological traits are similar to those of other known species of *Deuterophlebia*.

**Figure 5:**
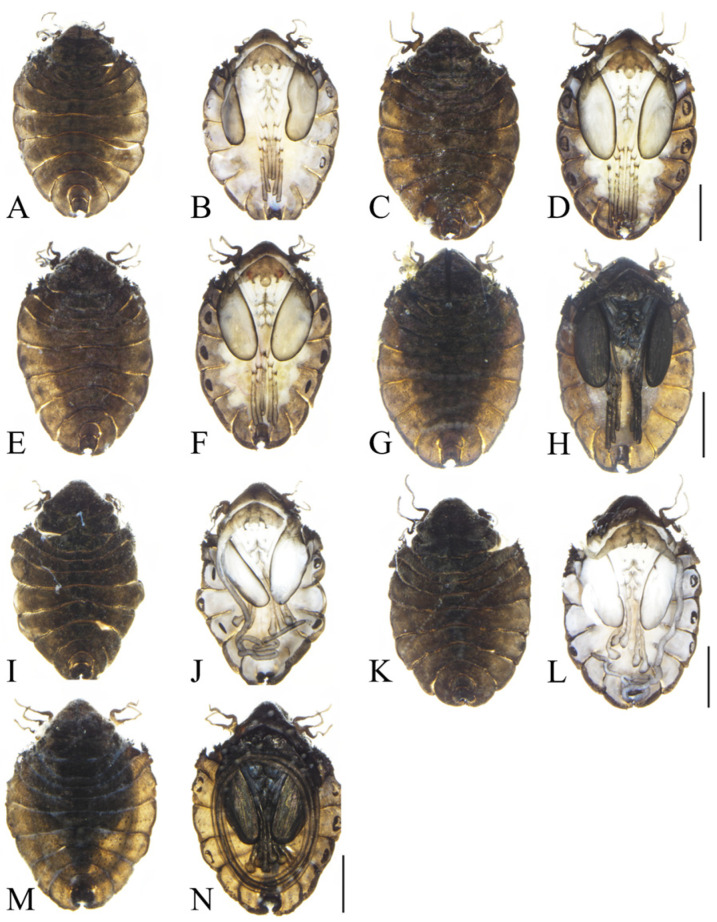
Morphological changes in the pupal period of *D. shawanensis* sp. nov.: (**A**) (dorsal view); (**B**) (ventral view); (**C**) (dorsal view); (**D**) (ventral view); (**E**) (dorsal view); (**F**) (ventral view); (**G**) (dorsal view); (**H**) (ventral view); (**I**) (dorsal view); (**J**) (ventral view); (**K**) (dorsal view); (**L**) (ventral view); (**M**) (dorsal view); (**N**) (ventral view); (**A**–**H**) Female pupae; (**I**–**N**) Male pupae. (**A**–**F**,**I**–**L**) young pupae; others are mature pupae. Scale bars = 1.0 mm.

We also found pupae at different stages of maturity in the samples collected from the family Deuterophlebiidae, such as young pupae and mature pupae, as shown in Figure 5. There is no great difference between young pupae and mature pupae on the dorsal view, but on the ventral view the young pupae have a tarsal limb and pseudopodia (white membranous) structure that the larvae have not completely degenerated. For the young pupae the color of their compound eyes is pale pink, and the color of the head is pale cinnamon. The color of the wings is white, surrounded by a circle of brownish-black edges, and the thorax is white. There are three pairs of furrows in the middle, the color of which is dark brown. The female antennal sheath is more concentrated, less scattered, and light brown, and the antennal sheath of the male has not yet formed into an oval annular structure, which is more chaotic than that of the mature male pupae. For the mature pupae, the color of the head, compound eyes, chest, wings, and antennae sheath of the mature pupae is black, and a shininess can be seen on the wings.

## 4. Discussion

We first found the larvae of *D. shawanensis* sp. nov. in October 2024, and a large number of pupae were collected in the following November and December. Unfortunately, there was a large amount of ice and snow on the road to the collection site during the February and March of 2025, which meant we could not carry out sampling work. Furthermore, no larvae, pupae, and adults were found at the collection site during the following seven months. Fortunately, we collected larvae of different ages and young pupae in their habitat in mid-August this year, but their distribution was small and may be in the early stages of development. Thus, we assumed that they had all emerged in the middle of March or in early April. In this case, we hypothesized that the eggs of *D. shawanaensis* sp. nov. hatch between August and September every year, and then the larvae develop into pupae from August to December. And in March of the next year, after around three months of hibernation, the pupae eclosion adults when the temperature rises, which also means that the development period of eggs is quite long, being more than six months. Kitakami (1938) hypothesized that there might be parthenogenesis in *D. nipponica* [4,17], while in *D. wuyiensis*, no males were found at all [4]; this likely represents a highly effective survival and reproductive strategy developed through evolution, showing strong environmental adaptability and diversity in reproductive tactics [18]. In the present study, the sex ratio of the pupal stage is about 2:1, indicating sexual reproduction.

For species morphological identification of Deuterophlebiidae, the pupal stage was considered as the most suitable stage [4]. The species collected in this study were not accompanied by a pair or pairs of spines at the pupal stage, which was different from the other Chinese species that had been described [4,13], and also morphologically different from the species which were collected in Japan and Kashmir, India [6,17]. However, it does have some particular similarities in morphology to *D. mirabilis*, which was collected in the Tianshan Mountains of Xinjiang (type species) by two foreign scholars in the early twentieth century [19], being also found in Kashmir, India [6]. As shown in Figure 3 and Figure 5, it was observed that the bulge of the dorsal plate of the head of the species in this study was more pronounced, and the small spines of the second thoracic segment were long on the small protrusions on both sides, which were significantly wider than the third thoracic segment. According to the hand-drawn illustrations of the scholar Brodsky (1930) [19], the protrusion of the dorsal plate of the head of *D. mirabilis* was not prominent, there was no small convex structure on both sides of the second thoracic segment, and the second thoracic segment was shorter than the third thoracic segment. The morphological differences among the already known species in Deuterophlebiidae are quite large and also some of the known species lack molecular biology data [4,13]. In this case, further study on species identification and classification of Deuterophlebiidae should focus on both sides; it is like that more new species will be discovered in the near future. Up to now, China has been the country with the highest species diversity worldwide [4,13].

Some scholars have proposed that the netting Blephariceridae family and the Deuterophlebiidae family should be classified into one category [20], but with the emergence of more advanced molecular sequencing methods, this statement is likely to be disproved [13,21]. The molecular differences between these two families are quite large, meaning they are independent of each other [13]. Morphologically, the larvae of the Blephariceridae family can attach to stones in turbulent streams, as they have six structures similar to “suckers” in the abdomen, allowing them to adhere tightly to rocks and to not easily to fall off [22]. Comparatively, the Deuterophlebiidae do not possess a “sucker” structure; despite not having this structure, they can also adhere to stones and are not easily dislodged due to their larvae having seven pairs of pseudopods with rows of hooked spines that assist in attaching to smooth stones in the water [5].

**Figure 6:**
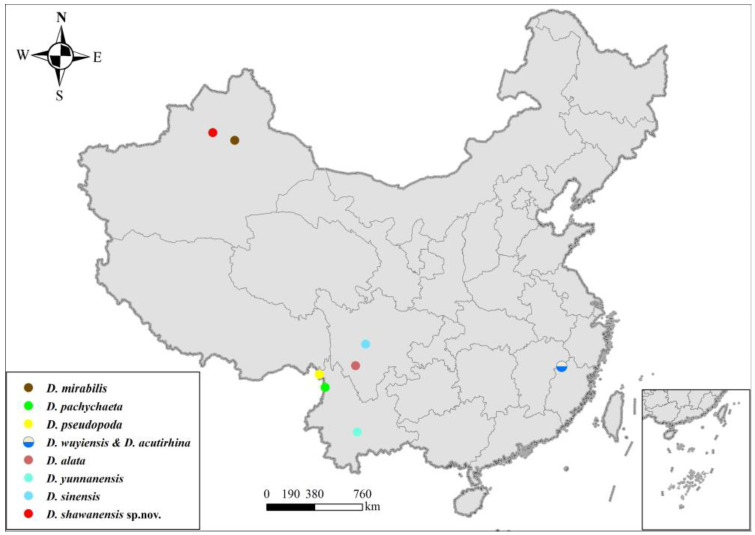
Distribution of Chinese *Deuterophlebia* spp.

Based on the global distribution map of Deuterophlebiidae species (Figure 6, modified from Courtney 1994 and Zheng et al., 2022, 2023) [4,5,13], the distribution of Deuterophlebiidae species is more concentrated in the Himalayan region. Considering biogeography, the Tianshan region of Shawan (the collection site of the new species in this study) can be regarded as part of the Himalayas [23]. There have been seven species discovered in the Himalayan region, which are *D. mirabilis*, *D. blepharis*, *D. brachyrhina*, *D. oporina*, *D. pseudopoda*, and *D. pachychaeta* [4,5], and also the new species in this study. Additionally, *D. brachyrhina* and *D. oporina* are considered sister species to the rest of the Deuterophlebiidae species, and their distribution is also limited to the Himalayan region [13]. This suggests that the Himalayas might be another distribution center of Deuterophlebiidae species, just like the Nearctic realm, where six Deuterophlebiidae species have been reported [4,5]. Compared to the Nearctic realm, the Himalayas and its surrounding areas have a unique climate and geographical environment, and thus, there might be undiscovered Deuterophlebiidae species in this area.

## 5. Keys to Asian Deuterophlebia Pupae

Modified from the key by Zheng et al. [4,15]. It contains nine known species with previously described pupal stages and one new species from this work.

1. Mesothorax with lateral outgrowths........................................................................*D. alata*

– Mesothorax without lateral outgrowths (Figure 2)...............................................................................................................................................2

2. Abdominal tergites VI–VII with posterolateral projections........................*D. pseudopoda*

– Abdominal tergites VI–VII without posterolateral projections (Figure 3)...............................................................................................................................................3

3. Mesothorax without spines on anterolateral margin (Figure 2)...............................................................................................................................................4

– Mesothorax with spines on anterolateral margin............................................................................................................................................6

4. have transverse chitin bands and a macrogranulation on the tergite...........*D. nipponica*

– without transverse chitin bands and a macrogranulation on the tergite.......................................................................................................................................5

5. Head backboard highly elevated, the second abdominal segment protrudes outwards with small spines, significantly longer than the third thoracic segment (Figure 2 and Figure 5)................................................................................................*D. shawanensis* sp. nov.

– Head backboard not highly elevated, the second abdominal segment does not protrude outward, shorter than or equal in length to the third thoracic segment........................................................................................................................*D. mirabilis*

6. Mesothorax with one pair of spines on anterolateral margin............................................................................................................................................7

– Mesothorax with two pairs of spines on anterolateral margin............................................................................................................................................8

7. Abdominal tergites with dark bands................................................................*D. bicarinate*

– Abdominal tergites without dark bands..................................*D. sajanica, D. yunnanensis*

8. Abdominal tergites with dark bands................................................................*D. wuyiensis*

– Abdominal tergites without dark bands.......................................................................................................................................9

9. Abdominal tergites with a pair of large dark dots..........................................*D. acutirhina*

– Abdominal tergites without obvious larger dark dots.........................................................................................................................................10

10. Mesothoracic spines expanded basally.........................................................*D. pachychaeta*

– Mesothoracic spines not expanded basally........................................................................................................................................11

11. Gills with elongated posterior filaments.............................................................*D. sinensis*

– Gills with indistinct posterior filaments........................................................*D. tyosenensis*

## Figures and Tables

**Table 1:** GenBank accession numbers of COI sequences and other information of specimens used in molecular study.

Species	Specimen Code	Life Stage	Sample Sites	GenBank Accession Number
*D. shawanensis* sp. nov.	XJ1	Female pupa	43°59′0.99″ N, 85°13′43.79″ E 1594.5 m	PX091279
	XJ2	Male pupa		PX091280

**Table 2:** Values of K2P genetic distance among the DNA barcodes (COI).

Values	*D. shawanensis* sp. nov.	*D. pseudopoda*	*D. pachychaeta*	*D. alatus*	*D. wuyiensis*	*D. acutirhina*	*D. sinensis*
*D. pseudopoda*	0.134						
*D. pachychaeta*	0.125	0.119					
*D. alatus*	0.143	0.138	0.143				
*D. wuyiensis*	0.143	0.139	0.150	0.140			
*D. acutirhina*	0.134	0.142	0.146	0.134	0.137		
*D. sinensis*	0.113	0.117	0.108	0.138	0.155	0.156	
*D. yunnanensis*	0.122	0.116	0.086	0.146	0.149	0.150	0.149

**Table 3:** Hydrological data of the site of collection.

Altitude (m)	Wide (m)	Deep (m)	T (°C)	COD (mg/L)	PH
1594.5	1.5	1.2	10	11	8.8

## Data Availability

The original contributions presented in this study are included in the article. Further inquiries can be directed to the corresponding author (guowei612@xjau.edu.cn (W.G.)).

## Abbreviation

The following abbreviation is used in this manuscript:

*D.*	*Deuterophlebia*
