# Correction: Wang et al. Description of a New Species of Mountain Midges (Diptera, Deuterophlebiidae) from Xinjiang, China. *Insects* 2025, *16*, 965

**DOI:** 10.3390/insects16121229

**Published:** 2025-12-04

**Authors:** Xin Wang, Minghui Gao, Xinyang Li, Rui Han, Jiayang Feng, Wei Guo

**Affiliations:** Xinjiang Key Laboratory for Ecological Adaptation and Evolution of Extreme Environment Organisms, College of Life Sciences, Xinjiang Agricultural University, Urumqi 830000, China; 18146913651@163.com (X.W.); gmhxxzj@xjau.edu.cn (M.G.); 15599773075@163.com (X.L.); lip546686@163.com (R.H.); 18030472729@163.com (J.F.)

## 1. Figure Legend

In the original publication [1], there was a mistake in the legend for “**Figure 3.** Male pupae of *D. shawanensis* sp. nov.: (**A**). Thoracic spine (dorsal view); (**B**). Gill (ventral view); (**C**). Posterior end (ventral view). Scale bars = 0.1 mm.”. We misinterpreted the legend in Figure 3A. We apologize for this. The correct legend appears is presented as follows: “**Figure 3.** Male pupae of *D. shawanensis* sp. nov.: (**A**). The small spines (ventral view); (**B**). Gill (ventral view); (**C**). Posterior end (ventral view). Scale bars = 0.1 mm.” We display the same direction for this species.

The authors state that the scientific conclusions are unaffected. This correction was approved by the Academic Editor. The original publication has also been updated.

## 2. Additional Contribution

A correction has been made to Section 3.1, Paragraphs 2–4 have been added as below:

**Type material**: Regarding the holotype of Deuterophlebiidae *Edw.* in the first published article on the proposed family of Deuterophlebiidae [16], there are significant morphological differences compared to the species we discovered. Firstly, the male and female pupae are shorter than our specimens. Secondly, the second thoracic segment is not prominently raised and is longer than the third thoracic segment, and the gill filament length is also shorter than that of our specimens. Our specimens have no spines on the dorsal side of the head capsule, so there are clear morphological differences from the holotype (Figures 5 and 6 in Ref. [16] and Figure 2C,D). Additionally, morphological comparisons with other species are also discussed in the article. Our type materials are preserved at Xinjiang Key Laboratory for Ecological Adaptation and Evolution of Extreme Environment Organisms, College of Life Sciences, Xinjiang Agricultural University.

**Type locality**: Regarding the collection sites of our specimens, we already indicated the latitude, longitude, and elevation of the sampling locations in the article, as well as other hydrological data at the time of sampling. We believe we have described them very accurately. For specific location information, please refer to Tables 1 and 3 and Figure 4 in the article.

**Type material deposition place**: The sampled rivers include a variety of substrate types such as silt, coarse sand, and gravel, and the surrounding habitats consist of forests, grasslands, and shrublands.

**Figure 2:**
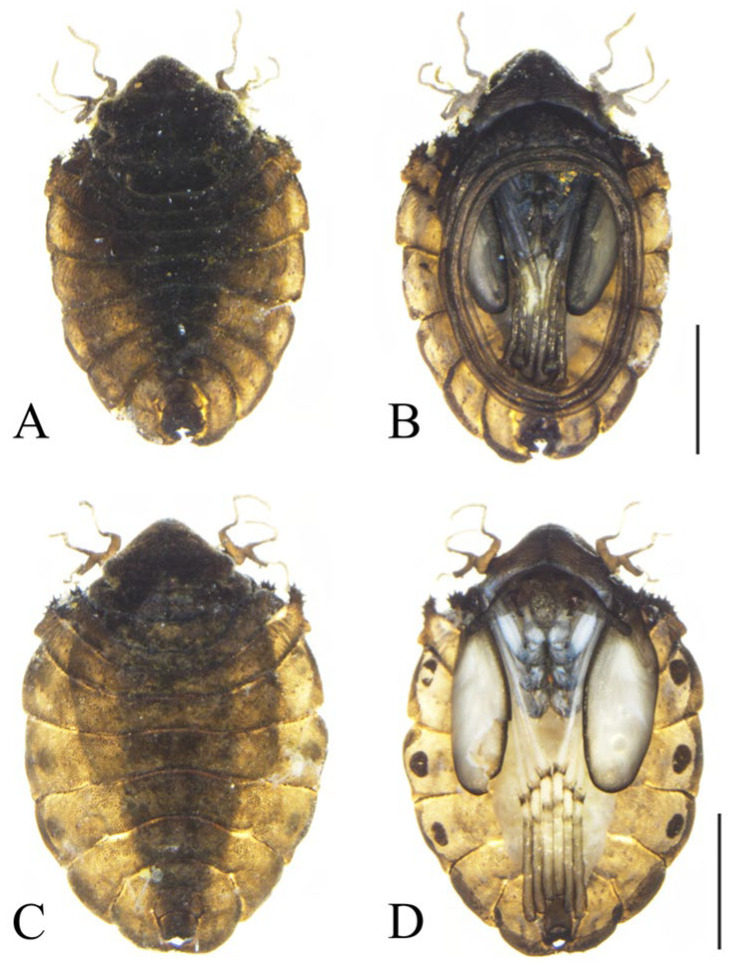
Pupae of *D. shawanensis* sp. nov.: (**A**). Male pupa (dorsal view); (**B**). Male pupa (ventral view); (**C**). Female pupa (dorsal view); (**D**). Female pupa (ventral view). Scale bars = 1.0 mm.

## 3. Additional Reference

16. Pulikovsky, N. Metamorphosis of *Deuterophlebia* Sp. (Diptera, Deuterophlebiidae *Edw.*). *Trans. R. Entomol. Soc. Lond.* **1924**, *72*, 45–62. https://doi.org/10.1111/j.1365-2311.1924.tb03350.x.

With this correction, the order of some references has been adjusted accordingly. The authors state that the scientific conclusions are unaffected. This correction was approved by the Academic Editor. The original publication has also been updated.
